# Increased Serum Levels of sCD14 and sCD163 Indicate a Preponderant Role for Monocytes in COVID-19 Immunopathology

**DOI:** 10.3389/fimmu.2020.560381

**Published:** 2020-09-23

**Authors:** Jose Gómez-Rial, Maria José Currás-Tuala, Irene Rivero-Calle, Alberto Gómez-Carballa, Miriam Cebey-López, Carmen Rodríguez-Tenreiro, Ana Dacosta-Urbieta, Carmen Rivero-Velasco, Nuria Rodríguez-Núñez, Rocio Trastoy-Pena, Javier Rodríguez-García, Antonio Salas, Federico Martinón-Torres

**Affiliations:** ^1^Grupo de Investigación en Genética, Vacunas, Infecciones y Pediatría, Instituto de Investigación Sanitaria de Santiago, Hospital Clinico Universitario and Universidade de Santiago de Compostela, Servizo Galego de Saúde, Galicia, Spain; ^2^Laboratorio de Inmunologìa, Servicio de Análisis Clìnicos, Hospital Clìnico Universitario Santiago de Compostela, Servizo Galego de Saúde, Galicia, Spain; ^3^Translational Pediatrics and Infectious Diseases Section, Department of Pediatrics, Hospital Clìnico Universitario de Santiago de Compostela, Galicia, Spain; ^4^Unidade de Xenética, Instituto de Ciencias Forenses, Facultade de Medicina, Universidade de Santiago de Compostela, and GenPoB Research Group, Instituto de Investigación Sanitaria (IDIS), Hospital Clìnico Universitario de Santiago, Servizo Galego de Saúde, Galicia, Spain; ^5^Intensive Medicine Department, Hospital Clìnico Universitario de Santiago de Compostela, Galicia, Spain; ^6^Pneumology Department, Hospital Clìnico Universitario de Santiago de Compostela, Galicia, Spain; ^7^Microbiology Department, Hospital Clìnico Universitario de Santiago de Compostela, Galicia, Spain; ^8^Clinical Biochemistry Laboratory, Hospital Clìnico Universitario de Santiago de Compostela, Galicia, Spain

**Keywords:** COVID-19, monocyte, sCD14, sCD163, immunopathology

## Abstract

**Background:**

Emerging evidence indicates a potential role for monocytes in COVID-19 immunopathology. We investigated two soluble markers of monocyte activation, sCD14 and sCD163, in COVID-19 patients, with the aim of characterizing their potential role in monocyte-macrophage disease immunopathology. To the best of our knowledge, this is the first study of its kind.

**Methods:**

Fifty-nine SARS-Cov-2 positive hospitalized patients, classified according to ICU or non-ICU admission requirement, were prospectively recruited and analyzed by ELISA for levels of sCD14 and sCD163, along with other laboratory parameters, and compared to a healthy control group.

**Results:**

sCD14 and sCD163 levels were significantly higher among COVID-19 patients, independently of ICU admission requirement, compared to the control group. We found a significant correlation between sCD14 levels and other inflammatory markers, particularly Interleukin-6, in the non-ICU patients group. sCD163 showed a moderate positive correlation with the time lapsed from admission to sampling, independently of severity group. Treatment with corticoids showed an interference with sCD14 levels, whereas hydroxychloroquine and tocilizumab did not.

**Conclusions:**

Monocyte-macrophage activation markers are increased and correlate with other inflammatory markers in SARS-Cov-2 infection, in association to hospital admission. These data suggest a preponderant role for monocyte-macrophage activation in the development of immunopathology of COVID-19 patients.

## Introduction

Emerging evidence from SARS-Cov-2 infected patients suggests a key role for monocyte-macrophage in the immunopathology of COVID-19 infection, with a predominant monocyte-derived macrophage infiltration observed in severely damaged lungs (1), and morphological and inflammation-related changes in peripheral blood monocytes that correlate with the patients’ outcome (2). An overexuberant inflammatory immune response with production of a cytokine storm and T-cell immunosuppression are the main hallmarks of severity in these patients (3). This clinical course resembles viral-associated hemophagocytic syndrome (VAHS), a rare severe complication of various viral infections mediated by proinflammatory cytokines, resulting in multiorgan failure and death (4). A chronic expansion of inflammatory monocytes and over-activation of macrophages have been extensively described in this syndrome (5–7). Viral-associated hemophagocytic syndrome has been identified as a major contributor to death of patients in past pandemics caused by coronaviruses (8), including previous SARS and MERS outbreaks (9), and currently suggested for SARS-Cov-2 outbreak (10).

CD14 and CD163 are both myeloid differentiation markers found primarily on monocytes and macrophages, and detection of soluble release of both in plasma is considered a good biomarker of monocyte-macrophage activation (11, 12). Elevated plasma levels of soluble CD14 (sCD14) are associated to poor prognosis in VIH-infected patients, are a strong predictor of morbidity and mortality (13, 14), and associated with diminished CD4+-T cell restoration (15). In addition, soluble CD163 (sCD163) plasma levels are a good proxy for monocyte expansion and disease progression during HIV infection (16). In measles infection, a leading cause of death associated with increased susceptibility to secondary infections and immunosuppression, sCD14 and sCD163 levels have been found to be significantly higher, indicating an important and persistent monocyte-macrophage activation (17).

We hypothesized that monocytes/macrophages may be an important component of immunopathology associated to SARS-Cov-2 infection. In this paper, we analyze serum levels of soluble monocyte activation markers in COVID-19 patients and their correlation with severity and other inflammatory markers.

## Materials and Methods

### Subjects

We recruited 59 patients with confirmed PCR-positive diagnosis of SARS-Cov-2 infection, classified according to ICU admission requirement (*n* = 22 patients), or non-ICU requirement (*n* = 37), and age-matched healthy individuals (*n* = 20) as a control group. Demographic data, main medication treatment and routine lab clinical parameters including inflammatory biomarkers were collected for all infected patients. Leftover sera samples from routine analytical controls were employed for the analysis, after obtaining the corresponding informed consent. Time elapsed from hospital admission to sample extraction was also recorded.

### Measurement of sCD14 and sCD163 Serum Levels

To determine levels of soluble monocyte activation markers in serum specimens, appropriate sandwich ELISA (Quantikine, R&D systems, United Kingdom) were used following manufacturer indications. Briefly, diluted sera samples were incubated for 3 h at room temperature in the corresponding microplate strips coated with capture antibody. After incubation, strips were washed and incubated with the corresponding Human Antibody conjugate for 1 h. After washing, reactions were revealed and optical density at 450 nm was determined in a microplate reader. Concentration levels were interpolated from the standard curve using a four-parameter logistic (4-PL) curve-fit in Prism8 GraphPad software. Final values were corrected applying the corresponding dilution factor employed.

### Statistical Analysis

Data are expressed as median and interquartile range. All statistical analyses were performed using the statistical package R. Mann–Whitney tests were used for comparison between ICU and non-ICU groups *versus* healthy controls. Pearson’s correlation coefficients were used to quantify the association between sCD14 and sCD163 concentration and other lab parameters in non-ICU patients. Data outliers, falling outside the 1.5 interquartile range, were excluded from the statistical analysis. The nominal significance level considered was 0.05. Bonferroni adjustment was used to account for multiple testing.

## Results

### Demographic and Clinical Laboratory Parameters

Patients in the ICU group showed significant differences when compared to non-ICU group in several clinical laboratory parameters: lymphocytes, ferritin, D-dimer, Lactate dehydrogenase (LDH), procalcitonin (PCT), and Interleukin-6 (IL-6). The absolute value for circulating monocytes did not show significant differences between groups. However, these values may have been distorted by the use of tocilizumab, an IL-6 blocking drug extensively employed in the ICU group which interferes with monocyte function. Age and time elapsed from admission to sample extraction did not show differences between groups. Values are summarized in Table 1.

**TABLE 1:** Demographic and clinical laboratory parameters of patients recruited.

Parameter	ICU	non-ICU	*P*-value
*Clinical laboratory parameters*			
Lymphocytes	0.54 (0.47–1.058)	1.16 (0.79–1.62)	**0.0004**
Monocytes	0.35 (0.16–0.65)	0,42 (0.35–0.58)	ns
Platelets	264 (204.3–354.5)	272 (213–413)	ns
D-Dimer	3676 (1198–8121)	755 (413–1033)	**0.0002**
Lactate dehydrogenase (LDH)	677 (429–818.5)	469 (391–595)	**0.0188**
C-reactive protein (CRP)	7.37 (2.56–20.51)	4,65 (2.16–11.41)	ns
Procalcitonin (PCT)	0.22 (0.09–0.4)	0.09 (0.05–0.21)	**0.0305**
Ferritin	1257 (837.3–3020)	467 (254.5–785)	**<0.0001**
Interleukin-6 (IL-6)	83.10 (14.45–381.8)	12.70 (6.95–46)	**0.0014**
Glycosylated hemoglobin (Hb1Ac)	5.95 (5.65–6.47)	6.1 (5.7–6.9)	ns
Troponin-I	0.021 (0.017–0.246)	0.017 (0.017–0.019)	ns
*Time elapsed from admission to sample (days)*			
	5 (3.75–10)	4 (2–6)	ns
*Age (years)*			
	52 (48.75–61.25)	52 (44–65)	ns
*Corticoids*			
	19/22 (87%)	2/37 (5.4%)	**<0.0001**

*Bold values are significant values.*

### Serum Levels for sCD14 and sCD163

**FIGURE 1:**
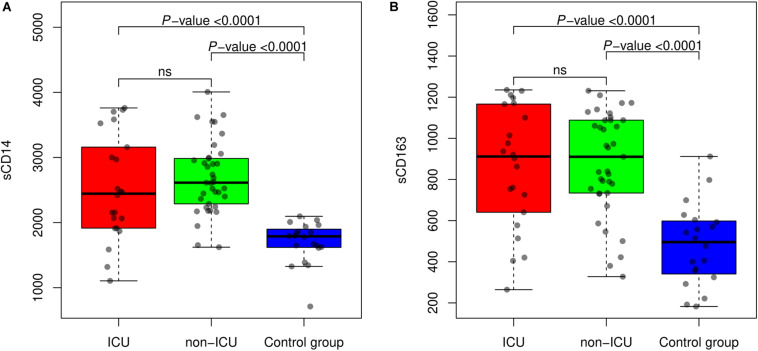
Values of sCD14 **(A)** and sCD163 **(B)** in sera samples from patients in ICU, non-ICU, and healthy controls. Results are presented as median and interquartile range levels in ng/ml. Non-parametric Mann–Whitney tests were used for comparison between groups, and *P*-values for the different comparisons are displayed.

Median levels for sCD14 in sera from ICU patients were 2444.0 (95%CI: 1914.0–3251.0) ng/ml, compared to 2613.0 (95%CI: 2266.0–2991.0) ng/ml in non-ICU patients. The healthy control group median value was 1788.0 (95%CI: 1615.0–1917.0) ng/ml. We observed significant statistical differences when comparing infected patients against controls (*P*-value < 0.0001), however no significant differences were observed between ICU and non-ICU groups. Median levels for sCD163 in sera from ICU patients were 911.5 (95%CI: 624.7–1167.0) ng/ml, and 910.4 (95%CI: 733.1–1088.0) ng/ml in non-ICU patients. The healthy control group value was 495.6 (95%CI: 332.5–600.7) ng/ml. As with sCD14, we observed significant differences for values from infected patients compared to control group (*P*-value < 00001), but no differences between ICU and non-ICU infected patients. Values are summarized in Table 2 and Figure 1.

**TABLE 2:** Concentration (ng/ml) of serum levels of sCD14 and sCD163 in patients from ICU and non-ICU groups, and healthy controls.

Concentration	ICU	non-ICU	Healthy controls
sCD14	2444.0 (1914.0–3251.0)	2613.0 (2266.0–2991.0)	1788.0 (1615.0–1917.0)
sCD163	911.5 (624.7–1167)	910.4 (733.1–1088)	495.6 (332.5–600.7)

*Data are represented as median and interquartile range.*

### Correlation Between sCD14 and sCD163 Levels and Time Elapsed From Hospital Admission

**FIGURE 2:**
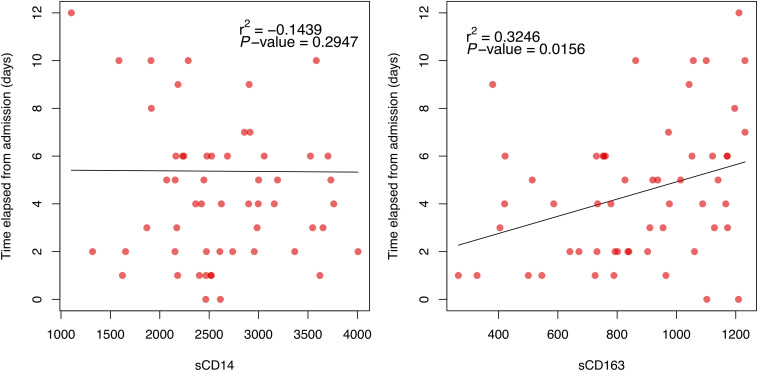
Correlation between serum levels of sCD14 and sCD163 and time elapsed from admission to sample extraction (in days) for all infected patients. Pearson’s correlation coefficient (*r*^2^) and *P*-value are shown.

We assessed the correlation between sCD14 and sCD163 levels and time elapsed from hospital admission to sample extraction (Figure 2). We found a significant positive correlation between sCD163 levels and time elapsed (*r*^2^ = 0.3246, *P*-value = 0.0156) We did not observe a significant correlation between sCD14 levels and time elapsed from hospital admission to sample extraction.

### Correlation Between sCD14 and sCD163 Levels and Clinical Laboratory Parameters

**FIGURE 3:**
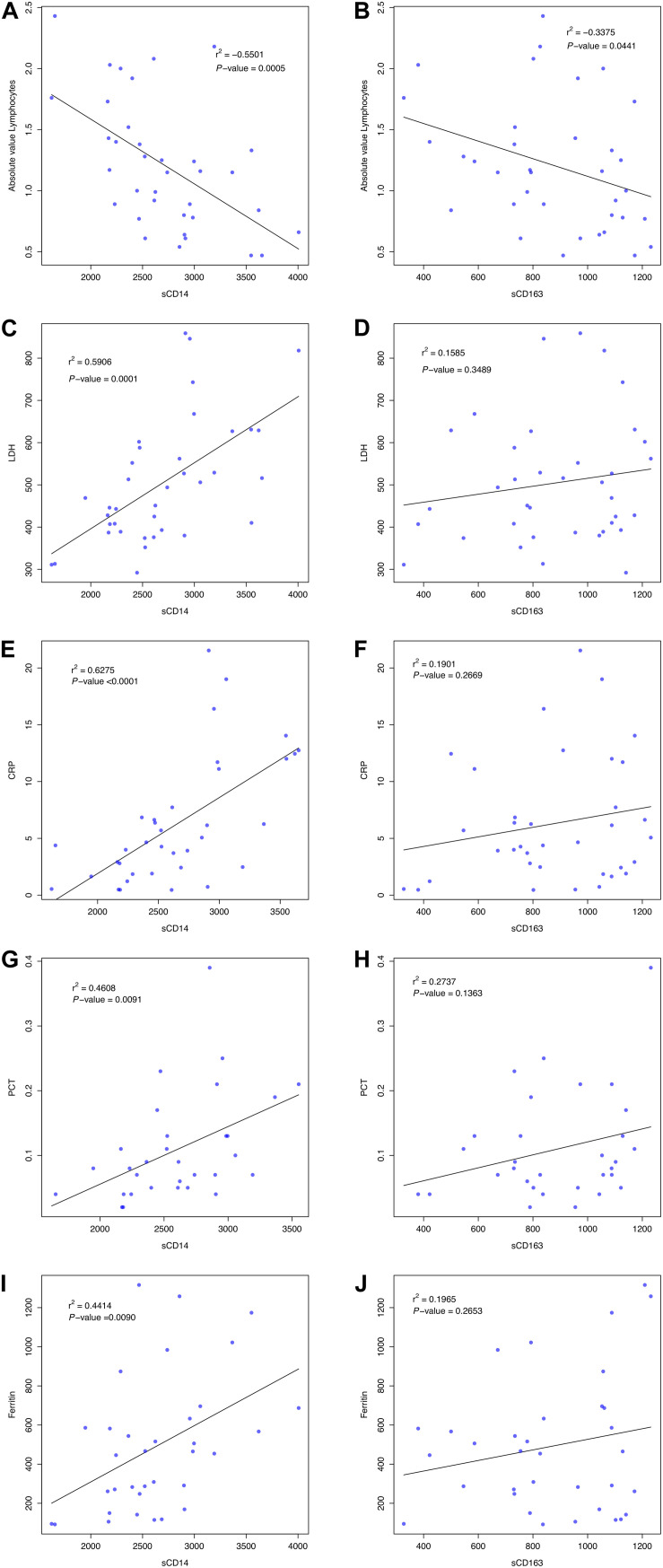
Association between serum levels of sCD14 and sCD163, and several laboratory parameters including Absolute Valor Lymphocytes **(A,B)**, LDH **(C,D)**, CRP **(E,F)**, PCT **(G,H)**, and Ferritin **(I,J)** in the non-ICU patient group. Pearson’s correlation coefficient (*r*^2^) and *P*-value are shown. LDH, lactate dehydrogenase; CRP, C-reactive protein; PCT, procalcitonin.

**FIGURE 4:**
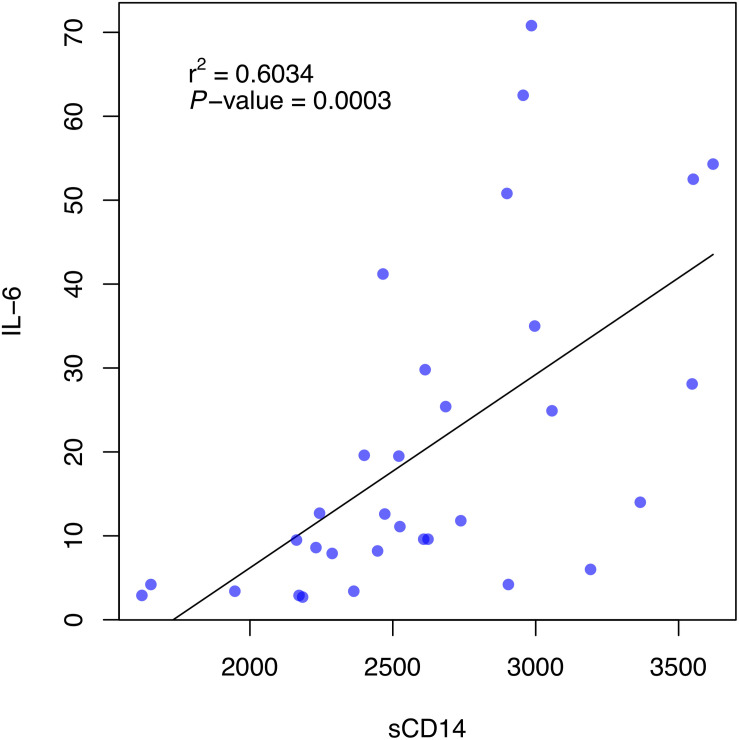
Association between serum levels of sCD14 and IL-6 levels in the non-ICU patient group. Pearson’s correlation coefficient (*r*^2^) and *P*-value are presented.

We found significant correlations between sCD14 and sCD163 levels and several clinical laboratory parameters in infected patients (in these analysis, adjusted significance under Bonferrori correction is 0.01), but only in the non-ICU group, possibly reflecting an interference of the use of tocilizumab or corticoids in the ICU group. Levels of sCD14 showed a negative correlation with the absolute value of lymphocytes (*r*^2^ = −0.5501, *P*-value = 0.0005) and a positive correlation with levels of LDH (*r*^2^ = 0.5906, *P*-value = 0.0001), CRP (*r*^2^ = 0.6275, *P*-value < 0.0001); PCT (*r*^2^ = 0.4608, *P*-value = 0.0091), and Ferritin (*r*^2^ = 0.4414, *P*-value = 0.0090) (Figure 3). No other significative associations were found with other lab parameters. Levels of sCD163 did not show significant correlation with clinical laboratory parameters (Figure 3). Particularly, IL-6 also showed significant positive correlation with sCD14 (*r*^2^ = 0.6034, *P*-value = 0.0003) (Figure 4).

### Effect of Treatment on sCD14 and sCD163 Levels

**FIGURE 5:**
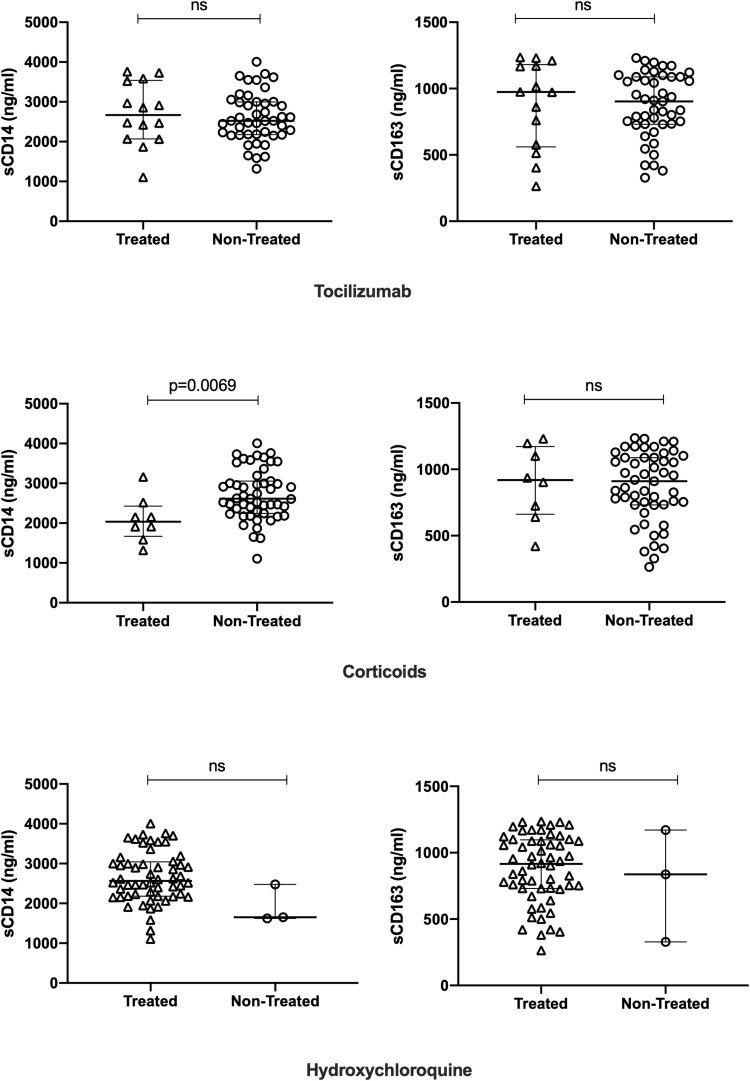
Effects of corticoid, hydroxychloroquine, and tocilizumab treatment on sCD14 and sCD163 levels. Results are presented as median and interquartile range levels in ng/ml. Non-parametric Mann–Whitney tests were used for comparison between groups, and *P*-values for the different comparisons are displayed.

We analyzed possible interference of different treatments on sCD14 and sCD163 serum levels for all patients. We found an interference of corticoid treatment on sCD14, levels with median values of 2034 (95%CI: 1319–3159) ng/ml for treated group, and values of 2613 (95%CI: 2466–2913) ng/ml for non-treated group. Values were significantly lower in corticoid-treated group (*P*-value = 0.0069) (Figure 5). No impact was found for corticoids on sCD163 levels. Likewise, hydroxychloroquine and/or tocilizumab were not found to have an impact on sCD14 and sCD163 serum levels.

### Correlation Between sCD14 and sCD163 Levels and Hospital Stay

Levels of sCD14 and sCD163 did not show association with length of hospital stay in both groups. Also, these biomarkers did not show association with the number of days of onset of symptoms.

### Age-Dependence of sCD14 and sCD163 Levels

We analyzed for possible age-dependence of sCD14 and sCD163 levels. Values did not show association between these biomarker levels and the age of patients.

## Discussion

Our results show, for the first time, increased levels of sCD14 and sCD163 in sera from SARS-Cov-2 infected patients admitted to hospital. We did not observe statistical differences when comparing ICU versus non-ICU patients. This is probably due to the interference on monocyte function and sCD14 levels produced by the use of corticoid treatment in ICU patients, as shown here and previously by others (18, 19). However, levels of sCD14 showed a strong correlation with clinical laboratory parameters, including acute phase reactants (ferritin, LDH, C-reactive protein, procalcitonin) and a strong correlation with IL-6 levels in the non-ICU patient group, where no corticoids treatments were used. Hydroxychloroquine and tocilizumab treatment did not show interferences on sCD14 and sCD163 levels. Furthermore, sCD163 levels showed a correlation with the time elapsed from hospital admission to sample extraction, suggesting a potential indicator of disease progression.

Monocytes and macrophages constitute a key component of immune responses against viruses, acting as bridge between innate and adaptive immunity (20). Activation of macrophages has been demonstrated to be pivotal in the pathogenesis of the immunosuppression associated to several viral infections (such as VIH, measles), where expansion of specific subsets of monocytes and macrophages in peripheral blood are observed, and considered to be drivers of immunopathogenesis (21). Our results support the hypothesis of a preponderant role for monocytes in SARS-Cov-2 immunopathology, associated to an overexuberant immune response. Increased levels of monocyte-macrophage activation markers, and their correlation with other inflammatory biomarkers (particularly IL-6), indicate a close relationship between monocyte activation and immunopathology in these patients. Inflammatory markers are closely related to severity in COVID-19 pathology (22) and selective blockade of IL-6 has been demonstrated to be a good therapeutic strategy in COVID-19 pathology (23). Our results thus suggest that monocyte-macrophage activation can act as driver cells of the cytokine storm and immunopathology associated to severe clinical course of COVID-19 patients. Further, monitorization of monocyte activity trough these soluble activation markers and/or follow-up of circulating inflammatory monocytes in peripheral blood, could be useful to assess disease progression in the same way as in other viral infections (16).

In addition, our results identify monocyte-macrophage as a good target for the design of therapeutic intervention using drugs that inhibit monocyte-macrophage activation and differentiation. In this sense, anti-GM CSF inhibitor drugs, currently under clinical trials for rheumatic and other auto-inflammatory diseases, might provide satisfactory results in COVID-19 patients. Other drugs targeting monocyte and/or macrophage could also be useful in COVID-19, as in other inflammatory diseases (24). The strategy of inhibiting monocyte differentiation has proved useful in avoiding cytokine storm syndrome after CAR-T cell immunotherapy (25), suggesting a possible therapeutic application to COVID-19 immunopathology (26, 27).

The present study has several limitations, including a relatively low sample size and the interference of corticoids in ICU patients’ results. However, these preliminary results are strongly suggestive of an important implication of monocyte-macrophage in COVID-19 immunopathology, as highlighted by the correlations found between these biomarker levels and inflammatory parameters. Further studies using broader series are needed to confirm our findings.

In summary, our data underscore the preponderant role of monocyte and macrophage immune response in COVID-19 immunopathology and provide pointers for future interventions in drug strategies and monitoring plans for these patients.

## Data Availability Statement

The raw data supporting the conclusions of this article will be made available by the authors, without undue reservation.

## Ethics Statement

The studies involving human participants were reviewed and approved by Comité de Ética de la Investigación con Medicamentos de Galicia (fast-track approval 18-march-2020). Written informed consent to participate in this study was provided by the participants’ legal guardian/next of kin.

## Author Contributions

JG-R, FM-T, and AS designed and conceptualized the study and made the first draft. MC-T, IR-C, AG-C, MC-L, CR-T, AD-U, CR-V, NR-N, RT-P, and JR-G collected the samples and did the analysis, reviewed the draft and approved the final version. All authors contributed to the article and approved the submitted version.

## Conflict of Interest

The authors declare that the research was conducted in the absence of any commercial or financial relationships that could be construed as a potential conflict of interest.
